# Vaccine Adjuvants: from 1920 to 2015 and Beyond

**DOI:** 10.3390/vaccines3020320

**Published:** 2015-04-16

**Authors:** Alberta Di Pasquale, Scott Preiss, Fernanda Tavares Da Silva, Nathalie Garçon

**Affiliations:** 1 GSK Vaccines, Avenue Fleming, 1300 Wavre, Belgium; E-Mails: scott.s.preiss@gsk.com (S.P.); fernanda.tavares@gsk.com (F.T.D.S.); 2 Bioaster, 321 Avenue Jean Jaurès, 6700 Lyon, France; E-Mail: nathalie.garcon@bioaster.org

**Keywords:** vaccine, adjuvant, safety, immunogenicity, immune response, innate immune response, adaptive immune response

## Abstract

The concept of stimulating the body’s immune response is the basis underlying vaccination. Vaccines act by initiating the innate immune response and activating antigen presenting cells (APCs), thereby inducing a protective adaptive immune response to a pathogen antigen. Adjuvants are substances added to vaccines to enhance the immunogenicity of highly purified antigens that have insufficient immunostimulatory capabilities, and have been used in human vaccines for more than 90 years. While early adjuvants (aluminum, oil-in-water emulsions) were used empirically, rapidly increasing knowledge on how the immune system interacts with pathogens means that there is increased understanding of the role of adjuvants and how the formulation of modern vaccines can be better tailored towards the desired clinical benefit. Continuing safety evaluation of licensed vaccines containing adjuvants/adjuvant systems suggests that their individual benefit-risk profile remains favorable. Adjuvants contribute to the initiation of the innate immune response induced by antigens; exemplified by inflammatory responses at the injection site, with mostly localized and short-lived effects. Activated effectors (such as APCs) then move to draining lymph nodes where they direct the type, magnitude and quality of the adaptive immune response. Thus, the right match of antigens and adjuvants can potentiate downstream adaptive immune responses, enabling the development of new efficacious vaccines. Many infectious diseases of worldwide significance are not currently preventable by vaccination. Adjuvants are the most advanced new technology in the search for new vaccines against challenging pathogens and for vulnerable populations that respond poorly to traditional vaccines.

## 1. The Evolution of Vaccines

The history of vaccination extends as far back as a millennium. Records suggest that the Chinese used inoculation (or “variolation”) techniques against smallpox as early as 900 AD [1]. Inoculation was based on the observation that those who survived smallpox were immune for life, and involved introducing dried pus, vesicular fluid or scabs from infected individuals into the skin or nasal cavity of healthy persons. While effective in inducing protective immunity, inoculation resulted in severe disease and death in a percentage of recipients. Edward Jenner is attributed as being the first to demonstrate through experimentation that vaccination could protect from disease without transmitting the disease itself. This was achieved in the late 18th century by taking advantage of the cross-protective effects of clinically mild cowpox infection in preventing smallpox. Since these first attempts human vaccines targeting several dozen viral and bacterial pathogens of global significance have been developed and used in clinical practice, and many more investigational vaccines continue to be designed and tested [2]. Vaccination is one of the most successful public health interventions ever implemented, and continues to have vast impacts in preventing disease and death due to infectious disease worldwide [3].

The key principle underlying immunization is the induction of an immune response capable of providing specific protection from infection or disease, and where the risk of acquiring the disease from vaccination has either been reduced or removed [4]. The vaccinated individual is rendered immune to disease on future exposure, and unwanted side-effects from the pathogen-induced disease are avoided. Early vaccines were live-attenuated or whole-pathogen preparations [4,5]. Attenuation is achieved by desiccation or by repeated passage in culture such that the virulence of a pathogen is reduced/removed but the organism remains viable [4]. Whole-pathogen preparations contain inactivated pathogens, initially achieved using exposure to high temperatures. While several live-attenuated and whole-pathogen vaccines continue to be used in the 21st century, some of these vaccines have historically faced difficulties in terms of reactogenicity or in achieving sufficient potency and efficacy [6]. Furthermore, the potential for reversion to virulence of live-attenuated vaccine strains, and incomplete inactivation of bacteria or viruses contained in vaccines, has occasionally caused cases of disease after vaccination, temporarily eroding confidence in these approaches [4].

The early 20th century saw several advances: the identification of bacterial toxins that could be modified to non-toxic forms while retaining high immunogenicity; the use of cell culture for bacterial and virus propagation and attenuation; and the commencement of the first systematic, national, vaccination programs as we know them today. The introduction of vaccination, improved hygiene, advancement in medicines and improved access to health care in many countries was accompanied by marked decreases in morbidity and mortality due to infectious diseases. As a result, in some settings, reactogenicity and serious adverse reactions attributed to vaccination (sometimes without causation being demonstrated) were no longer considered acceptable by the general public. In the 1970s public confidence in vaccination plummeted when whole-cell pertussis vaccines were erroneously linked to the onset of encephalitis in children. Pertussis vaccination coverage dropped precipitously in some countries (such as the United Kingdom), and vaccination against pertussis was halted in others (Sweden), resulting in national pertussis outbreaks of a magnitude not seen for decades [7,8]. The response of the scientific community was to search for purified antigens (or sub-units) capable of inducing a protective immune response and with improved reactogenicity profiles [9]. The resulting acellular pertussis vaccines containing between one and five purified antigens demonstrated lower rates of local and systemic reactions after vaccination compared with whole-cell vaccines [10]. Nevertheless, the duration of immunity induced by acellular pertussis vaccines appears to be shorter than expected [11,12,13], underlining the need for regular booster doses in older children as well as in adolescents, adults and the elderly. At the same time, the search continues for improved pertussis vaccines that induce more durable protection [14].

Other new vaccine approaches were developed to address a range of technical and implementation-related challenges. For example, recombinant technologies allowed the production of vaccines for pathogens unable to be grown *in vitro*. Pathogens with multiple disease-causing strains/serogroups required methods to combine multiple antigens into a single vial. Efforts were also made to improve vaccine acceptance and coverage using complex multi-valent vaccines targeting multiple different diseases in the same injection.

**Figure 1:**
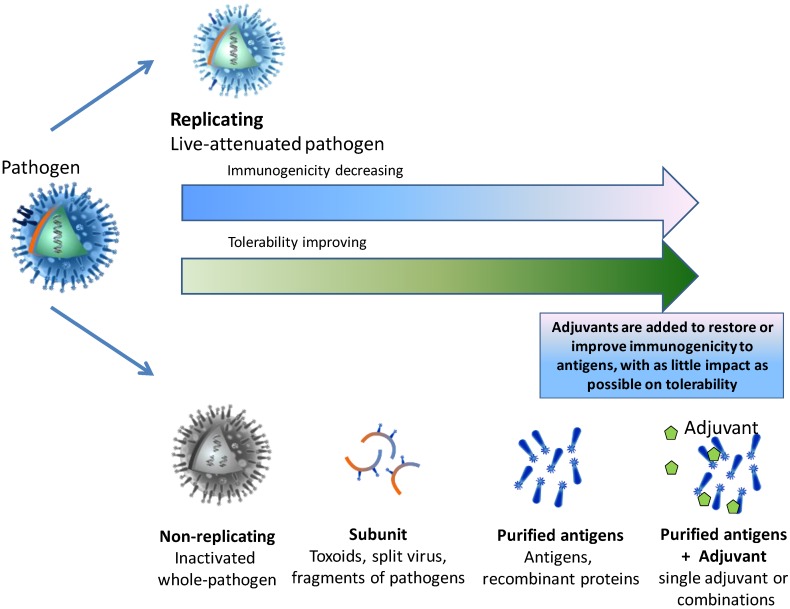
Balancing immunogenicity and tolerability.

While vaccines containing a limited number of purified antigens generally have improved safety profiles compared with live-attenuated and whole-pathogen vaccines, they are also often less immunogenic due to the removal of pathogenic features of the organism (Figure 1) [15].

Improving the anticipated benefits (efficacy) of immunization while decreasing their potential risks (adverse reactions) underpins the development of all new vaccines and is a key factor driving new technologies and sophisticated vaccine design. Thus, rather than containing whole pathogens (live attenuated or inactivated) many modern vaccines contain purified antigen in suspension with preservatives or stabilizers, as well as possible trace substances left over from the manufacturing process, such as egg proteins, antibiotics, or formaldehyde. Many vaccines also contain an adjuvant or adjuvant combination: these are substances added to vaccines specifically because of their immune enhancing effects. The word “adjuvant” means “to help/aid”, and adjuvants were initially used to counter the poor immunogenic potential of highly purified antigens. In recent years their role has expanded as our understanding of the immunology of vaccination has grown.

### The Immunology of Infection and Immunization

Intense investigation of immunological mechanisms means that we are closer to understanding the processes involved in the identification and clearance of pathogens from the human body and in establishing immune memory to respond to future exposure. This information has implications for vaccine design, allowing identification of specific immunogens capable of stimulating immune responses that lead to protection.

**Figure 2:**
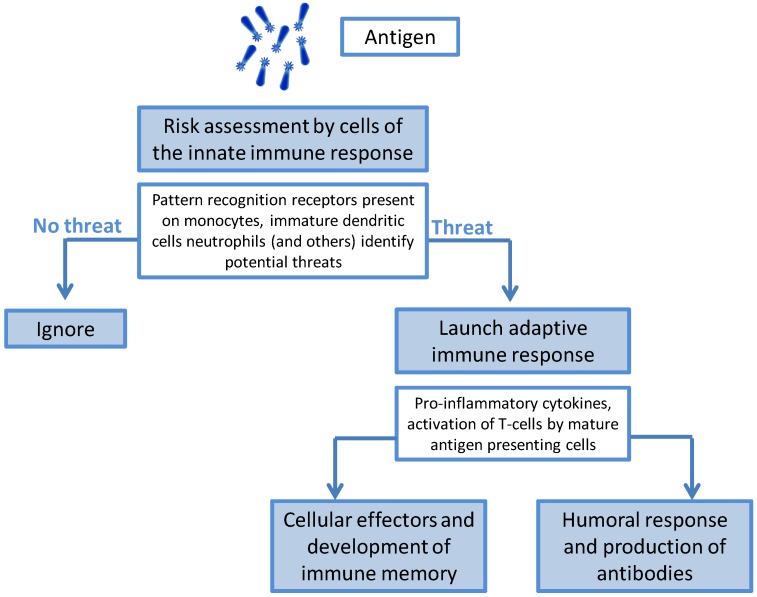
The role of the innate immune response.

The immune response to an infectious agent (or to immunization) can be broadly divided into two phases: the innate and adaptive responses [16]. When exposure to foreign matter occurs, cellular effectors of the innate immune response, such as macrophages, monocytes, neutrophils and dendritic cells, are able to recognize specific surface patterns (pathogen-associated molecular patterns or PAMPS) using pattern recognition receptors (PRRs) that classify the agent as a threat or as benign [17] (Figure 2).

Recognition of a potential pathogen sets off a complex series of events that can include phagocytosis, release of inflammatory mediators including chemokines and cytokines, activation of complement and cellular recruitment; all of which may lead to the development of signs and symptoms of local inflammation in the individual. Antigen taken up by innate cells, such as dendritic cells, is processed, with cellular differentiation into APCs. The APVs migrate to the T-cell region of the draining lymph node, where the link between the innate and adaptive immune response occurs. Innate immune responses have no capacity for memory. The development of immune memory is an adaptive response and will only occur if the correct signals are given by the effectors of the innate response. Thus, the manner by which the innate immune response sets in motion the secondary or adaptive immune response has profound implications for the type of secondary response, the quality of the response and the induction of immunological memory.

The adaptive immune response is largely driven by lymphocytes: T-cells and B-cells. The relative activities of the B-cell and T-cell populations determine the type of immune response generated in response to infection. On recognizing a specific antigen, B-cells differentiate into plasma cells and release specific antibodies (IgM) into the circulation. However, the development of immune memory or the ability to respond rapidly on re-exposure to the same antigen only occurs when B-cells have received T-cell “help”—a so-called “T-cell dependent response”. CD4+ T-helper cells are unable to recognize antigen unless it is presented to them after processing by the antigen-presenting cells (APCs) activated during the innate immune response. Activated T-helper cells release inflammatory mediators that are specific to a T-helper cell sub-population (Th1, Th2, Th17 and Thf [18,19]), which has downstream implications for how effectively the pathogen is removed or contained. In broad terms Th1 cells are needed for the removal of intracellular pathogens; Th2 cells for the removal of extracellular parasites; Th17 for removal of bacteria and fungi; and Thf cells for activating a T-cell dependent B-cell response [18]. Th1 and Th17 cells are also mediators of autoimmunity, whereas Th2 cells are associated with asthma and allergic diseases. Activated CD8+ cytotoxic T-cells can kill cells directly or through the release of cytotoxins.

Current knowledge suggests that APCs, such as dendritic cells, play a key intermediary role between the innate and adaptive responses and are critical in determining the direction of the adaptive immune response [16]. The ideal vaccine, therefore, would initiate an innate immune response capable of directing the adaptive immune response toward efficient inactivation and removal of the specific pathogen, followed by the development of immune memory. The limited ability of highly purified vaccines to induce protective immunity appears to be related to their failure to induce maturation of APCs [16].

Past experiences illustrate how manipulating the immune response can overcome the limitations of purified vaccines. Purified polysaccharide vaccines are poorly immunogenic and largely ineffective in infants due to immune immaturity and the inability of polysaccharide to induce T-cell responses [20]. Linking (or conjugating) polysaccharide to a protein carrier alters the manner in which the innate cells present antigen, stimulating the induction of T-helper cell maturation. A T-cell dependent response follows, with the development of mature antibodies and immune memory. By changing the immune response, protein-conjugate vaccines revolutionized the prevention of meningitis and severe bacteremia due to *Haemophilus influenzae*, *Neisseria meningitidis* and *Streptococcus pneumoniae* in infants who had previously been unable to be protected.

On the other hand, early attempts to develop a vaccine against respiratory syncytial virus (RSV) stalled for several decades after an experimental formalin-inactivated vaccine led to more severe RSV disease (with two deaths) in vaccinated children on re-exposure [21]. The heightened response on re-exposure was thought to be due to an excessively Th2-biased immune response to vaccination [22]. Since then, efforts continue to be directed towards the development of RSV vaccines capable of eliciting a response that does not lead to enhanced disease on re-exposure, and inducing prolonged protection [23].

The experience with conjugate vaccines and with RSV highlights the importance of understanding the type of immune response required to clear a specific pathogen. Technologies to identify and elicit specific immune responses are now available and continue to improve, and adjuvants are one means by which the immune system can be better directed.

## 2. The Discovery of Adjuvants

**Figure 3:**
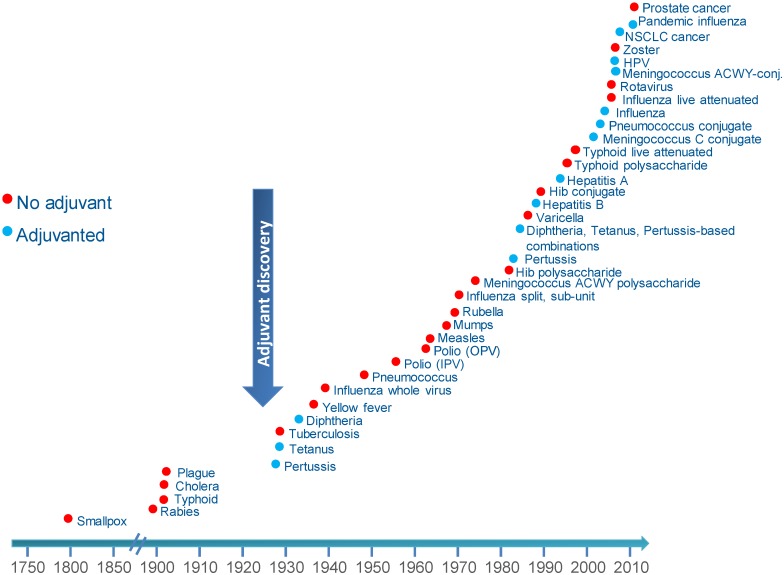
Licensed vaccines with or without adjuvant.

Like many important medical breakthroughs, the discovery of the immune-enhancing effects of adding an adjuvant to a vaccine was serendipitous. Gaston Ramon, a French veterinarian, observed that the yield of tetanus and diphtheria anti-sera from horses was higher from animals that had developed an abscess at the injection site [24]. By injecting starch, breadcrumbs or tapioca, he induced sterile abscesses at the site of injection with inactivated toxin, and thus was able to increase anti-sera production, confirming the hypothesis that substances able to induce local inflammation at the injection site were also able to enhance anti-sera yield. Around the same time, Alexander Glenny working with colleagues in London discovered the immune-enhancing effects of aluminum salts. Aluminum was first used in human vaccines in 1932 and was the only adjuvant in use in licensed vaccines for approximately 70 years. Despite its extensive and continuous use, the immune mechanism of action of aluminum remains incompletely understood [25]. Aluminum adjuvants act primarily to increase antibody production and are therefore suitable for vaccines targeting pathogens killed primarily by antibodies. Aluminum-adjuvanted vaccines have not been successful in preventing infection due to intracellular pathogens [25]. Another early adjuvant attempt was a mineral oil-in-water emulsion (Freund’s incomplete adjuvant) which was considered too reactogenic for continued use in humans. Adjuvants have been used for more than 90 years and are currently components of more than 30 licensed vaccines from different manufacturers (Figure 3).

Not all vaccines need adjuvants. Live-attenuated vaccines are effective because they induce mild infection in recipients, and an immune response that is very similar to that induced by infection with wild-type strains: *i.e.*, these vaccines are capable of initiating innate immunity, which drives subsequent adaptive responses that lead to successful clearance of the pathogen. Some inactivated whole-pathogen vaccines have been successful because they contain a heterogeneous mixture of diverse antigens and other pathogen components that act as intrinsic adjuvants. However, these types of vaccines are not suitable when natural infection itself does not convey long-standing immunity, or when the pathogen is unable to be grown in culture.

**Figure 4:**
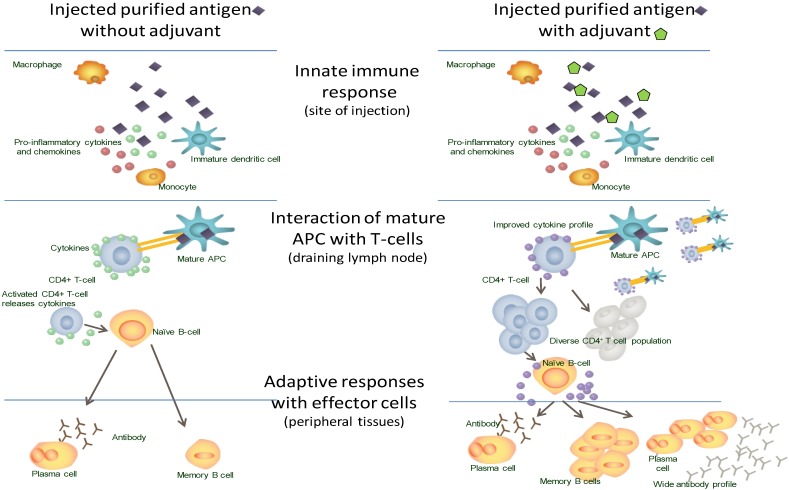
The immune response to vaccination with and without adjuvant.

Highly purified vaccine components frequently lack PAMPs, which means that the initial innate immune response is not activated such that an effective downstream adaptive response occurs. It is thought that the primary mechanism of action of adjuvants is on the innate immune response (Figure 4). Adjuvants can act like PAMPs, triggering the innate immune response through a variety of mechanisms, to identify the vaccine components as a “threat”, with activation and maturation of APCs and initiation of downstream adaptive immune activities [26].

## 3. New Vaccines: Challenges and Solutions

**Figure 5:**
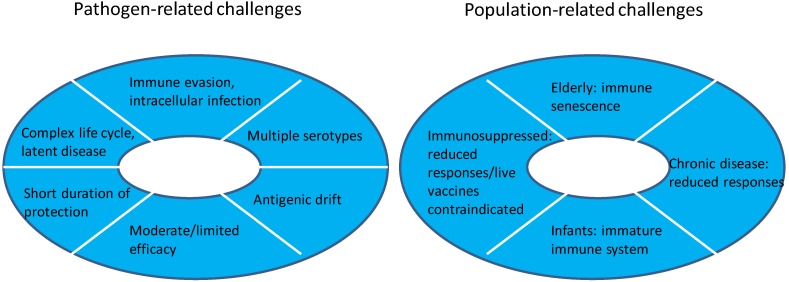
Challenges for modern vaccine development.

Many of the infectious diseases that historically caused widespread morbidity and mortality in young children have been largely controlled by effective vaccines. Previously common diseases such as diphtheria, tetanus, poliomyelitis, smallpox and measles were able to be prevented using relatively simple vaccines that stimulated robust antibody responses. Notable exceptions include malaria, tuberculosis and human immunodeficiency virus (HIV) infection. These pathogens have sophisticated mechanisms to evade human immune responses and are not able to be effectively prevented by antibodies alone. Hence despite the incredible success of vaccination as a public health intervention, infectious diseases remain the most common cause of death in children less than 5 years of age [27], while respiratory infections, diarrhea, HIV and tuberculosis all rank in the top ten leading causes of death in all age groups [28]. Thus, in the 21st century the technical challenges facing development of new vaccines are two-fold [4,29]: the first challenge relates to the characteristics of the pathogens themselves, and the second to the characteristics of the target populations (Figure 5).

### 3.1. Challenging Pathogens

Pathogens that make vaccine development challenging are those that are intracellular (for example, *Mycobacterium tuberculosis*), those with complex life-cycles (malaria), those that induce immune dysfunction in the host (HIV) and those with a latent disease phase (Herpes viruses, *M. tuberculosis*, and human papillomavirus (HPV)).

For some other challenging pathogens vaccines have been successfully developed and licensed for use, but their efficacy is sub-optimal and/or short-lived. For example, while pertussis vaccines have successfully reduced childhood deaths from pertussis, their short duration of protection (that parallels the inability of natural infection to provide life-long immunity) means that vaccinated individuals become susceptible over time. Pertussis infection, now more common in adolescents and adult populations, can be transmitted to young, highly vulnerable unvaccinated infants in whom disease can be very severe. Bacilli Calmette-Guérin (BCG) has been in use since the 1920s. BCG is effective in preventing severe, rapidly progressive tuberculosis in children but does not prevent infection or re-activation of latent disease, and has had little impact on tuberculosis disease control worldwide. Some bacteria, such as *S. pneumoniae* and *N. meningitidis*, have multiple disease-causing serotypes/serogroups whose epidemiology varies regionally and over time. Multi-component vaccines target the most common strains. Ideally improved vaccines capable of preventing disease from any strain would avoid the risk of type replacement and disease from a strain not contained in available vaccines. Many bacterial (*N. meningitidis*) and viral pathogens (HIV and influenza) undergo continuous evolution. In the case of influenza this process can be rapid, with new strains emerging from year to year, requiring new vaccines each influenza season.

### 3.2. Challenging Populations

Prevention of infectious disease in the elderly has become a priority in the 21st century as the proportion of older individuals increases globally. Increasing age is accompanied by negative impacts on the immune system that affect the functioning of innate and adaptive immune responses [30,31]. In clinical practice this translates to increased susceptibility to infection and reduced responses to traditional vaccines. Other populations at increased risk of severe infections and in whom traditional vaccines have reduced immunogenicity because of poor efficiency of all of the components of the immune system, include the immunocompromised, individuals with chronic disease, and neonates and infants (particularly premature infants).

### 3.3. Potential Solutions for New Vaccines

Multiple approaches exist to address the challenges of pathogen and population [4,32]. These developments have been enabled by expanding knowledge within the field of immunology, improved understanding in how the human immune system responds to pathogens and individual antigens at a molecular level, and improved understanding of the effects of immune deficiencies and of ageing on responses to immunization.

Novel vaccine design approaches include viral vector vaccines, whereby codes for specific peptides or antigen are inserted in the genome of modified viruses capable of infecting human cells. During viral multiplication and infection these peptides are recognized by the immune system as pathogenic signals and are processed by APCs with induction of adaptive immune responses. Viruses investigated for use as vectors include vaccinia virus (modified vaccinia virus Ankara), and adenovirus [33,34,35]. DNA vaccines are another novel vaccine design in which bacterial plasmid DNA is loaded with genes encoding the antigen of interest [36]. Expression of the antigen occurs once the plasmid is taken up into host cells. To date, DNA vaccines have shown limited immunogenicity in humans and their development has been slow to progress [37]. Dendritic cell vaccines constitute another novel approach and consist of infusions of antigen-treated mature, (usually autologous) dendritic cells that are infused back into the patient, and are currently used in the context of cancer therapy. No viral-vector or DNA vaccines have been licensed to date, although viral-vector vaccines have progressed to Phase III studies in humans.

The most advanced approach in terms of reaching licensure is that of the development of novel adjuvants. Greater understanding of the mechanisms of action of adjuvants and the specific immune requirements necessary to successfully prevent infection/disease due to individual pathogens now means that modern adjuvants can be more rationally selected to direct the immune system toward an effective response. Moreover, combinations of adjuvants can further modulate the required immune response in such a way as to result in more specific immune outcomes.

## 4. Modern Adjuvants

Modern adjuvants are being designed to overcome the pathogen and population-related challenges facing 21st century vaccines [32,38]. As such, sophisticated adjuvants may have the potential to help prevent infectious diseases of global significance for which successful vaccines have not been possible using traditional technologies.

A key adjuvant function is overcoming the poor immunogenicity of subunit vaccines by improving pathogen recognition and eliciting a response similar to the natural innate immune response. When effective, adjuvants can increase the breadth and durability of the response achievable using purified sub-unit antigen. The practical outcomes of the enhanced immune response are several: adjuvants can enable reductions in the quantity of antigen contained in individual vaccine doses. The improved quality of the immune response may mean that fewer vaccine doses are required to achieve immunity. The combined features of dose reduction and antigen sparing can have important implications for improving global vaccine supply. Adjuvants can also improve immune responses in populations where responses to vaccines are typically reduced, such as infants, the elderly and the immunocompromised.

By impacting the initiating signal to the innate immune system, the choice of adjuvant/s can direct the type of adaptive immune response to the administered antigen: preferentially activating specific T-cell responses. Based on published data for the AS04 and the AS03 adjuvants, their direct effects are on innate immune cells and effectors and not on adaptive mechanisms [39,40]. These effects are short-lived, and mostly limited to the site of injection and regional lymph nodes (Figure 4) [39].

The next step was the development of adjuvants containing more than one immune-stimulatory molecule (Adjuvant Systems). The proof-of-concept for Adjuvant Systems came with the development of GSK’s malaria vaccine. A suitable antigen (the recombinant RTS,S antigen targeting the pre-erythrocytic stage of the malaria parasitic life-cycle) was identified for vaccine development in the mid-1980s. Aluminum as adjuvant proved unsuccessful and pre-clinical and human challenge studies, were conducted to explore different combinations of immune-stimulatory molecules [41]. AS02 was initially selected, but during clinical development it became apparent that AS01 might provide additional benefits compared to AS02: RTS,S/AS01 induced higher cell-mediated immune responses and appeared to induce greater clinical protection in human challenge studies than RTS,S/AS02 [42,43]. The final candidate malaria vaccine tested in Phase III trials contains AS01, and vaccine efficacy against malaria infection has been demonstrated in vaccinated children [44].

The first vaccine to use an adjuvant other than aluminum was a hepatitis A vaccine licensed in the mid-1990s, which uses a virosome adjuvant system [45]. Virosomes are spherical phospholipid layers carrying bound influenza antigen on the surface or encapsulated within the lumen [46]. The virosome structure and components can be varied to direct the uptake and interaction with effectors of innate immune responses, impacting the initiation of downstream adaptive T-cell and B-cell activities.

In the last 20 years, six more adjuvants have been included in licensed vaccines (Table 1). The most commonly administered adjuvant after aluminum consists of oil-in-water emulsions using oils with improved reactogenicity compared to Freund’s original adjuvant. Several oil-in-water emulsions use squalene, a naturally occurring and readily metabolized oil. These emulsions induce robust humoral and cellular immune responses [47]. Some adjuvant systems contain combinations of adjuvants (Table 1) and have been specifically designed to increase T-cell immune responses.

The improvements in vaccine immunogenicity when antigen is administered with an adjuvant are exemplified by the case of H5N1 pandemic influenza vaccines. Unadjuvanted H5N1 influenza vaccines showed markedly lower immunogenicity than seasonal influenza strains [48]. Furthermore, sub-clade variants emerged rapidly after cases in humans started to be reported. Compared to unadjuvanted vaccines, adjuvanted H5N1 pandemic influenza vaccines induced improved immunogenicity in all age ranges and cross-reactive immunity against sub-clade variants and antigen sparing allowing increased supply [48]. The H5N1 experience confirmed the benefits provided by the addition of adjuvants, and the potential to address some of the influenza pandemic challenges.

As well as allowing improvements in pandemic influenza vaccines, new adjuvants have enabled the successful development of vaccines against challenging pathogens such as HPV. As HPV may remain undetected by the immune system, it is thought that high serum antibody levels are needed in order to achieve adequate concentrations at the cervix, where infection occurs. The HPV-16/18-AS04-adjuvanted vaccine was shown to induce significantly higher titers of HPV-specific antibodies and neutralizing antibodies than an aluminum-adjuvanted formulation [49]. The HPV-16/18-AS04-adjuvanted vaccine was licensed for use after conclusive demonstration of efficacy [50,51,52,53,54].

Adjuvants have also been used to improve immune responses in populations that may respond poorly to vaccination. For example, patients with end-stage renal disease tend to respond less well to aluminum-adjuvanted hepatitis B vaccines than healthy individuals, but have an increased risk of acute hepatitis B infection and progression to chronic hepatitis [55]. Compared to aluminum-adjuvanted hepatitis B vaccines, the licensed AS04-adjuvanted recombinant hepatitis B vaccine induces a higher and more durable antibody response, with enhanced cellular responses in patients with end-stage renal disease [56].

The expansion of our understanding of the potential role of adjuvants has brought about rapid advances in vaccine development. New adjuvanted vaccines under development target challenging pathogens such as dengue fever, cytomegalovirus infection and HIV, malignancies such as melanoma and lung cancer, and challenging populations such as the elderly [37]. Despite rapid progress in antigen and adjuvant design, knowledge gaps remain in our understanding of the immune system; such as the relative contribution of the innate and adaptive responses to protection against individual pathogens, and the precise mode of action of individual adjuvants. Information from a range of new disciplines such as systems biology, vaccinomics, transcriptomics and epigenetics may contribute to our understanding of vaccination and immunity in new ways. Deeper understanding of the mode of action of adjuvants will also facilitate evaluation of the safety profile of new adjuvanted vaccines.

**Table 1:** Characteristics of adjuvants used in licensed vaccines.

Adjuvant	Composition			Major Immune Effects
(vaccines where used)	**Component**	**Origin**	**Other Uses**	
**Aluminum** (D, T, pertussis, IPV, hepatitis A & B, HPV, meningococcal and pneumococcal)	Aluminum as salts mixed with antigen (adsorption)	Naturally occurring present in soil, water, air	Medicines, cosmetics, food industry	Increases local inflammation, improves antigen update by APCs. Acts to increase antibody production
**Virosomes** (Hepatitis and influenza)	Vesicles where influenza antigens in aqueous volume are enclosed within a standard phospholipid cell membrane bilayer	Natural phospholipids, Seasonal influenza glycoproteins	None	Increases uptake by APCs. May interact with B cells leading to T-cell activation.
**AS04** (Hepatitis B, HPV)	(3-deacyl-monophosphoryl lipid A) derived from LPS from *Salmonella Minnesota*, *Aluminum salts*	Natural exposure to LPS from Gram-negative bacteria occurs frequently	None	Directly stimulates TLR-4 increasing APC maturation and Th1 responses.
**MF59^®^** (Influenza-seasonal and pandemic)	Squalene	Animal source (shark liver oil). Found naturally in human tissues: adipose tissues, skin, arterial walls, skeleton, muscles, lymph nodes	Cosmetics, moisturizers	Increases APC recruitment and activation. Promotes antigen uptake and migration of cells to lymph nodes.
**AS03** (Influenza-pandemic)	Vitamin E (α-Tocopherol) Surfactant polysorbate 80 Squalene	Naturally occurring in humans. Surfactant and emulsifier Animal source (shark liver oil). See above	Vitamin Used in foods, eye drops & intravenous injections Naturally occurring. See above	Promotes local production of cytokines and recruitment of innate cells.
**Thermo-reversible oil-in-water** (Influenza-pandemic)	Squalene	Animal source (shark liver oil). See above	Naturally occurring. See above	Not reported
**ISA51** (therapeutic vaccine NSCLC)	Mineral oil DRAKEOL 6 VR Surfactant mannide-mono-oleate	Refined mineral oil of vegetable origin	Food industry	Strongly immunogenic

D = diphtheria, T = tetanus, IPV = inactivated poliomyelitis vaccine, HPV = human papilloma virus, LPS = lipopolysaccharide, APC = antigen presenting cells, TLR = toll-like receptor, NSCLC = non-small cell lung cancer, MPL = monophosphoryl lipid A.

## 5. Determining the Safety of Adjuvanted Vaccines

The safety of any adjuvant component is evaluated in the context of the vaccine in which it is used. That is, while vaccine components are tested individually early in development (preclinical phase), the bulk of safety assessment undertaken for any vaccine considers the final product.

**Figure 6:**
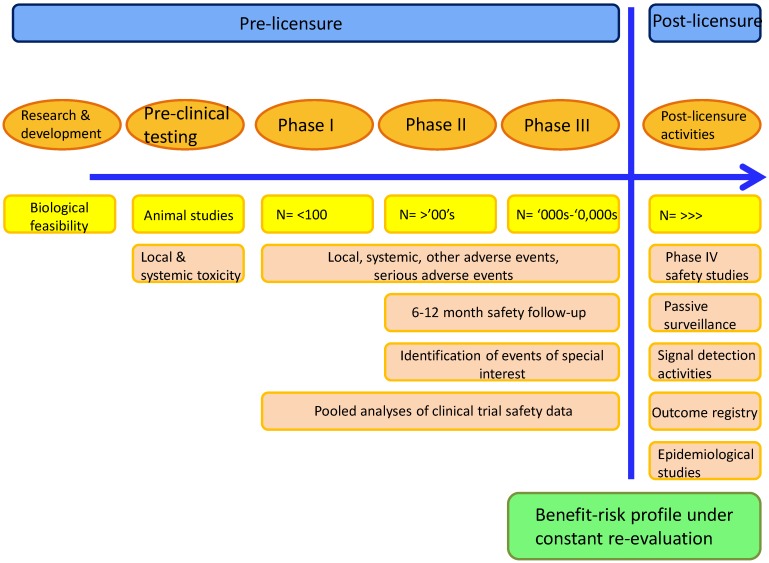
Characterization of safety from the laboratory to licensure and beyond

The evaluation of vaccine safety begins in the laboratory and continues indefinitely after licensure (Figure 6) [57,58]. Before administration to humans, vaccine candidates undergo rigorous testing in animal models designed to detect evidence of local or systemic toxicity that might indicate a potential safety issue in humans [59]. Where possible, tests to assess the effect of administering multiple doses, vaccine quality, immunogenicity and protective efficacy are all conducted in animal models prior to the first injection in humans. Pre-clinical tests for reproductive and developmental toxicology are also done if the vaccine is intended for women of childbearing age.

First-time-in-human (Phase I) vaccine studies are generally of small size and are conducted in healthy adults. These studies are frequently designed to reduce potential safety risks using strategies such as staggered enrolment, and dose-limiting toxicity that is pre-defined based on the results of animal testing. Phase II and III studies evaluate the candidate vaccine administered to ever increasing numbers of subjects, including the target population, with concurrent evaluation of safety and immunogenicity and/or efficacy endpoints. Independent Data Monitoring Committees whose role is to monitor safety outcomes in an unblinded manner may be used.

The most commonly encountered pre-licensure study design is the randomized, controlled trial, which allows for high probability to detect vaccine adverse effects compared to control, but they have limited statistical power to detect potential rare (1:10,000 to < 1:1000 doses) and very rare (<1:10,000) adverse events. Strategies such as pooling safety information from multiple, similarly designed studies may provide increased power to detect those rare adverse events. Vaccines come to licensure with a relatively extensive safety database allowing for a good understanding of the safety profile of the vaccine. However the acquisition of knowledge about the safety risks continues throughout the vaccine life cycle. Licensure of a new vaccine is based on the demonstration of its anticipated benefits in preventing disease that clearly outweigh any potential risk to the population to which it is targeted: this is referred to as the benefit-risk ratio. The benefit-risk of all vaccines remains under constant review, regardless of the presence of a novel adjuvant in the formulation.

### Assessment of the Benefit-Risk Ratio

The evaluation of the benefits of specific vaccines may change over time as the targeted disease comes under control, and may be reduced in some populations in which the vaccine is poorly efficacious. In the same way, the evaluation of risks of vaccination may change as new information on the safety profile of the vaccine come to hand, and may be different in some vulnerable populations (e.g., pregnant women, immunosuppressed individuals). Thus, a range of post-licensure activities around effectiveness and safety are planned prior to licensure to ensure that vaccine safety in different settings/populations is monitored and that appropriate actions are taken based on the results of these activities. In addition, pharmacovigilance processes are rigorously applied by regulatory authorities.

Despite the wealth of safety information that comes after licensure, the evaluation of potential safety signals that arise, in particular, the evaluation of studies attempting to establish a causal association with vaccination, can be complex and inconclusive. There are numerous examples where alleged causal relationships between vaccination and an adverse event have been shown to be unfounded. However, there have been instances in which vaccines have been withdrawn after licensure either due to demonstration of causal associations between the vaccine and an adverse event, or due to poor efficacy, that eventually changed the benefit-risk ratio [60,61]. When evidence arises from safety signal monitoring, or if new important adverse events are reported, actions are taken promptly by vaccine manufacturers and regulatory agencies, and the evidence is communicated rapidly to healthcare professionals and the public to allow informed decision-making about the most current benefit-risk profile.

## 6. Potential Safety Concerns around Adjuvanted Vaccines

Concerns about the safety of vaccination are not new, nor are these concerns specific to adjuvanted vaccines. Some of the concerns about adjuvanted vaccines are discussed in the following sections.

### 6.1. Reactogenicity

By counteracting the poor immunogenicity of pure antigen in some vaccines, the addition of adjuvants may lead to an increase in local reactions such as pain, redness, swelling at the site of injection and sometimes general symptoms such as fatigue, malaise, myalgia and fever [62,63]. Overall, the results of studies that have compared vaccines with and without adjuvant have shown a consistent trend toward increased reactogenicity, mainly at the injection site of the adjuvanted formulation [62,63]. The most frequently reported symptom is pain at the injection site. Overall the reported symptoms are mild-to-moderate in intensity, do not last for more than a few days, and do not have an impact on compliance with the vaccination schedule.

Observed reactogenicity of adjuvanted formulations may be a consequence of the enhanced activation of the innate immune response induced by adjuvant at the site of injection, which is expressed as a local inflammatory response. As with other vaccines, the reactogenicity profile of any adjuvanted vaccine is specific to the antigen and the target population studied [64,65,66,67]. Nevertheless, all licensed adjuvanted vaccines have shown a favorable benefit-risk ratio.

### 6.2. Immune-Mediated Diseases

Because adjuvants act directly as immune-stimulants there is a theoretical possibility that they may induce unwanted immune processes in the recipient that could trigger the onset of immune-mediated disease in susceptible individuals. Specific data collection methods including prolonged follow-up after vaccination have been devised to evaluate these adverse events of interest [68]. Efforts are ongoing to identify any increased risk of immune-mediated disease after vaccination with adjuvanted vaccines [69]. The available evidence, which includes pooled analyses of clinical trial data and post-licensure epidemiological studies of varying design, has generally not shown an increased risk in immune-mediated diseases associated with adjuvanted vaccines [70,71,72,73].

An example of an immune-mediated disease that has repeatedly been potentially linked with vaccination is Guillain-Barré syndrome (GBS). The concern that immunizations might trigger GBS in susceptible individuals initially arose after a small increase in the incidence of GBS was observed after “swine flu” vaccines were used in the United States in 1976 [74,75]. Subsequent studies showed only a slight-to-no increase in risk after seasonal influenza vaccination during later seasons [76,77].

In 2009, mass vaccination with new adjuvanted pandemic H1N1 influenza vaccines started in Europe. The potential risk of GBS for these new vaccines was unknown, prompting studies in Europe and internationally to assess the risk of GBS after vaccination with adjuvanted pandemic vaccines [78,79,80]. The results of these studies showed a non-statistically significant increase in GBS risk after vaccination, or an excess risk of one to three cases per million vaccinees, confirming the favorable benefit-risk profile of the vaccine.

Another example of an immune-mediated disease potentially linked to vaccination occurred in 2010, when a number of cases of narcolepsy following vaccination with *Pandemrix*™ (H1N12009/AS03, GlaxoSmithKline, Belgium) pandemic influenza vaccine were reported in some European Countries during the 2009 H1N1 influenza pandemic. Narcolepsy is a chronic neurological disorder caused by the brain’s inability to regulate sleep-wake cycles normally. It is a complex disease with a number of potentially contributing factors, including genetic and environmental factors, such as infections. The body of data accumulated suggests an increased risk of narcolepsy in individuals vaccinated with the vaccine *versus* the unvaccinated population. Further research is needed to better understand how other factors (genetic, environmental, circulating infections) associated with narcolepsy may have played a role. Other studies have been initiated to evaluate the biological plausibility by which vaccination may have triggered narcolepsy. In response, European Authorities, in collaboration with the vaccine manufacturer, promptly communicated the data gathered and regularly updated the vaccine label and the vaccine risk management plan. Authorities have also recognized that the benefit-risk profile of H1N12009/AS03 remains favorable, and have therefore recommended the maintenance of the marketing authorization [81].

### 6.3. Gulf War Syndrome 

Gulf War Syndrome comprises an ill-defined and varying group of systemic symptoms that occurred in veterans of the 1991 Persian Gulf War. The cause is unknown but links have been suggested with post-traumatic stress or exposure to chemicals and/or biological weapons or vaccination against anthrax [82]. An association was claimed between the presence of antibodies against squalene, an adjuvant used in the anthrax vaccine administered to soldiers, and the Gulf War Syndrome, based on the observation that antibodies to squalene were detected in the sera of most patients affected [82]. Further studies have subsequently shown that squalene was not present in vaccines administered to these soldiers. In addition, it is known that squalene is a component of the human body and low titers of anti-squalene antibodies are routinely found in healthy individuals [83]. WHO Safety Committee in 2006 concluded that fears that squalene in vaccine could induce pathological anti-squalene antibodies are unfounded [84].

### 6.4. Myofasciitis

In 1998, safety concerns about the use of aluminum in vaccines arose in France when deltoid muscle biopsies in patients with a constellation of symptoms including myalgia and fatigue, showed microscopic histological lesions called macrophagic myofasciitis (MMF). These lesions contained aluminum salts and where shown to persist for up to 10 years [85]. Because the MMF lesions occurred in the usual injection site in the deltoid, MMF was linked with the administration of aluminum-containing vaccines [86]. At that time, the World Health Organization (WHO) and the French Medicine Agency, in consultation with experts, encouraged animal and epidemiological studies specifically designed to investigate the issue. Studies in animals, patients with MMF and healthy individuals suggest that MMF represents a “vaccination tattoo” (a marker of prior vaccination) and that aluminum and microscopic inflammation may persist at the injection site in the long-term. To date, there are no reliable scientific data showing that this “vaccination tattoo” causes symptoms or other consequences [87,88]. Of note, the number of observed MMF cases is very small as compared to the millions of people who are vaccinated with aluminum-containing vaccines; information on the prevalence of MMF lesions in the healthy population are lacking; the symptoms reported by patients with MMF are non-specific and very common; and there is large variation in time elapsed between vaccination and symptom onset. A French study that reviewed the association between local MMF lesions and any generalized illness in the Cynomolgus monkey concluded that the persistence of aluminum-containing macrophages at the site of a previous vaccination was not associated with specific clinical symptoms or disease [87]. In 2008 the WHO Global Advisory Committee on Vaccine Safety (GACVS) issued a statement concluding that: “From the most recent evidence available, there is no reason to conclude that a health risk exists as a result of administration of aluminium containing vaccines, nor is there any good reason for changing current vaccination practice. The GACVS will continue to review the evidence that might emerge from on-going studies” [88].

## 7. Challenges around Implementing Vaccination Programs with Novel Vaccines

For many vaccines to be effective at a population level, high vaccine coverage is needed. Vaccine coverage relies on individuals being able to access and afford vaccination. In this regard, access and affordability of vaccination remain major hurdles to global vaccine coverage. UNICEF estimated that 1.5 million children alone died from vaccine-preventable disease in 2011 [89]. Many more deaths in adults from infection and cancer (cervical cancer as a result of HPV infection or liver cancer as a result of hepatitis B infection) are also vaccine-preventable.

Other issues around public confidence in vaccine safety and efficacy, trust in companies and agencies that manufacture or recommend vaccines and the changing perceptions around the need for vaccination, can all have significant impacts on vaccine uptake in specific populations [90,91]. Novel vaccine formulations, including novel adjuvants, are often at the center of discussions around possible safety concerns because the science underlying vaccine design and how vaccines work is not widely known. It is important for educators and experts in the field to communicate the need for new or improved vaccines, how they work to enhance the immune response and how they meet the highest quality and safety standards. It is also important to explain in simple terms the scientific principles underlying choice and development of vaccine formulations to increase understanding and acceptance. Manufacturers and public health agencies work towards meeting these challenges to make new adjuvanted vaccines readily available, and to communicate their value to all who may benefit from them.

## 8. Conclusions

Adjuvants have been used in vaccines for more than 90 years. Adjuvants were initially used in an empirical fashion to enhance the immune response to antigen, but became necessary components of many vaccines as purified antigens with lower immunogenicity were selected more and more frequently, as compared to live attenuated and whole-pathogen vaccine approaches. Our understanding of the potential of adjuvants to promote the activities of APCs and thus potentiate downstream adaptive immune responses is evolving. This information can enable the development of new vaccines targeting diseases against which older vaccine technologies were ineffective. The right match of antigens and adjuvants has a key role to play in these developments. New adjuvants have already contributed to more effective influenza vaccines, as well as vaccines targeting hepatitis B and HPV. A more rational selection of antigen and adjuvants could enable better protection of vulnerable populations that respond poorly to traditional vaccines, and may open the doors to new applications beyond prevention.
